# Synthesis, anti-angiogenic and DNA cleavage studies of novel *N*-(4-methyl-3-((4-(pyridin-3-yl)pyrimidin-2-yl)amino)phenyl)piperidine-4-carboxamide derivatives

**DOI:** 10.1186/s13065-017-0354-5

**Published:** 2017-11-30

**Authors:** Vinaya Kambappa, G. K. Chandrashekara, N. D. Rekha, Prasanna D. Shivaramu, Komaraiah Palle

**Affiliations:** 1 Department of Chemistry, Government First Grade College, Kadur, 577 548 India; 2 Department of Oncological Sciences, Mitchell Cancer Institute, USA Mitchell Cancer Institute, 1660 Springhill Avenue, Mobile, AL 36604 USA; 3 Department of Studies in Biotechnology, JSS College of Arts, Commerce & Science, Ooty Road, Mysore, 570 025 India; 4 Department of Nanotechnology, Visvesvaraya Technological University, Center for Postgraduate Studies, Bengaluru Region, Muddenahalli, Ckikkaballapur, 562 101 India

**Keywords:** Pyrimidine, 3-acetylpyridine, *N*-methyl morpholine, Antiangiogenic activity, CAM assay, DNA cleavage activity

## Abstract

A series of novel *N*-(4-methyl-3-((4-(pyridin-3-yl)pyrimidin-2-yl)amino)phenyl)piperidine-4-carboxamide derivatives **10**(**a**–**f**), **12**(**a**–**c**) and **14**(**a**–**c**) were synthesized and characterized by FTIR, ^1^H-NMR, mass spectral and elemental analysis. The efficacy of these derivatives to inhibit in vivo angiogenesis was evaluated using chick chorioallantoic membrane (CAM) model and their DNA cleavage abilities were evaluated after incubating with calf thymus DNA followed by gel electrophoresis. These novel piperidine analogues efficiently blocked the formation of blood vessels in vivo in CAM model and exhibited differential migration and band intensities in DNA binding/cleavage assays. Among the tested compounds **10a**, **10b**, **10c**, **12b**, **14b** and **14c** showed significant anti-angiogenic and DNA cleavage activities compared to their respective controls and the other derivatives used in this study. These observations suggest that the presence of electron donating and withdrawing groups at positions 2, 3 and 4 of the phenyl ring of the side chain may determine their potency and as anticancer agents by exerting both anti-angiogenic and cytotoxic effects
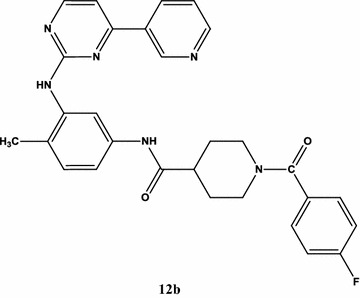
.

## Introduction

There is growing evidence that tumor-initiated neovascularization, called tumor angiogenesis, is a central process involved in the aggressive growth of tumors and of their metastases. The requirement of angiogenesis for sustained tumor growth has led to the development of alternative strategies for treating cancer based on the selective interference with the growth of tumor micro vessels [1]. Cancer, the second largest cause of mortality in the world, is continuing to be a major health hazard in developing as well as in developing countries [2]. Design and development of anticancer drugs with fewer or no side effects are important for the treatment of cancer. The search for such potential anticancer drugs has led to the discovery of synthetic molecules with anticancer activity.

DNA is an important drug target and it regulates many biochemical processes that occur in the cellular system. The different alleles present in the DNA are involved in various processes such as gene activation, gene transcription, mutagenesis, carcinogenesis etc. [3]. Many small molecules exert their anticancer activities by binding with DNA, thereby altering DNA replication and inhibiting the growth of tumour cells. DNA cleavage reaction is also considered of prime importance as it proceeds by targeting various parts of DNA such as purine and pyrimidine bases, deoxyribose sugar and phosphodiester linkage.

Small molecules that hydrolyze the DNA are useful in genetic engineering, molecular biotechnology and robust anticancer drug design [4, 5]. Heterocyclic compounds have emerged as potential therapeutic agents because of conformational rigidity, improved physical properties, charge density, lipophilicity and pharmacological advantages such as metabolic stability and oral bioavailability [6].

Pyrimidines and their analogues represent an important class of biologically active nitrogen containing heterocyclic molecules, and many of which are either available as natural compounds or by designed synthetic routes [7–9]. The pyrimidine derivatives comprise a diverse and interesting group of drugs and have been discussed [10–12]. Pyrimidine, being an integral part of DNA and RNA, have imparts diverse biological activity viz. anticancer [13, 14], antiviral [15, 16] antiprotozoal [17], antihypertensive [18], antihistaminic [19], anti-inflammatory [20], central nervous activities [21], antibacterial [22, 23], antifungal [24, 25] and in particular antiangiogenic agents [26]. Specifically, disubstituted pyrimidines have shown potent anticancer activity as CDK inhibitors [27], TNF-α inhibitors [28], Abl tyrosine protein kinase inhibitors [29], PI-3 kinase inhibitors [30], Akt kinase inhibitors [31], and cytokines inhibitors [32].

Imatinib, an anti-cancer agent prepared by an intermediate *N*-(5-amino-2-methylphenyl)-4-(3-pyridyl)-2-pyrimidinamine, and it is currently marketed as Gleevec. Imatinib selectively inhibits Bcr–Abl kinase and was first approved to treat both adult and children with Philadelphia chromosome-positive (Ph^+^) chronic myelogenous leukemia (CML) and later it has been approved to treat gastrointestinal stromal tumors (GISTs) [33] and other malignancies. Due to its high selectivity towards Bcr–Abl kinase, it has shown high efficacy and mild side effects in patients and has been listed as essential medicines by World Health Organization [34]. The use of combinatorial approaches toward the synthesis of drug-like scaffolds is a powerful tool in helping to speed up drug discovery. In the view of the facts mentioned above and as part of our initial efforts to discover potentially active new agents [35–37], we have synthesized some novel *N*-(4-methyl-3-((4-(pyridin-3-yl)pyrimidin-2-yl)amino)phenyl)piperidine-4-carboxamide derivatives as anticancer cancer agents, which have demonstrated efficient DNA binding and antiangiogenic activity.

## Materials and methods

### Chemistry

Melting points were determined using SELACO-650 hot stage melting point apparatus and were uncorrected. Infrared (IR) spectra were recorded using a Jasco FTIR-4100 series. Nuclear magnetic resonance (^1^H NMR) spectra were recorded on Shimadzu AMX 400-Bruker, 400 MHz spectrometer using DMSO-d6 as a solvent and TMS as internal standard (chemical shift in δ ppm). Spin multiplets are given as s (singlet), d (doublet), t (triplet) and m (multiplet). Mass and purity were recorded on a LCMSD-Trap-XCT. Silica gel column chromatography was performed using Merck 7734 silica gel (60–120 mesh) and Merck made TLC plates.

#### Synthesis of 3-dimethylamino-1-(pyridin-3-yl)prop-2-en-1-one (**3**)

A mixture of 3-acetylpyridine **1** (25 g, 20.63 mmol) and *N*,*N*-dimethylformamide dimethyl acetyl **2** (31.95 g, 26.82 mmol) was refluxed for 16 h under nitrogen. Upon completion of the reaction, the mixture was concentrated under reduced pressure. To the residue, cyclohexane was added and the mixture was cooled to 0 °C. The precipitate was collected by filtration to afford the product as yellow crystals (90%). MP: 78–80 °C. ^1^H-NMR (CDCl_3_) δ: 9.0 (d, 1H, Py-H), 8.62 (dd, 1H, Py-H), 8.25 (dt, 1H, Py-H), 7.81 (d, 1H, –COCH=CH), 7.35 (dd, 1H, Py-H), 5.75 (d, 1H, –COCH=CH), 3.25 (s, 3H, –CH_3_), 3.02 (s, 3H, –CH_3_). IR (KBr, cm^−1^): 3080, 1685, 1620, 1448, 1354, 748. MS (ESI) *m/z*: 177.09.

#### Synthesis of *N*-(2-methyl-5-nitrophenyl)-4-pyridin-3-yl-pyrimidin-2-ylamine (**5**)

To a mixture of 3-dimethylamino-1-(pyridin-3-yl)propenone **3** (25 g, 11.34 mmol) and *N*-(2-methyl-5-nitrophenyl)guanidinium nitrate **4** (47.66 g, 14.74 mmol) in *n*-butanol (200 mL), sodium hydroxide (8.63 g, 216 mmol) was added. The mixture was refluxed for 16 h and then cooled to 0 °C. The precipitate was collected by filtration and washed with methanol and diethyl ether and dried to get the product (92%) as a yellow solid. MP: 196–197 °C. ^1^H-NMR δ: 8.93 (d, 1H, Py-H), 8.71 (dd, 1H, Py-H), 8.60 (s, 1H, –NH), 8.45 (d, 1H, pyrimidyl-H), 8.30 (d, 1H, Py-H), 7.45 (dd, 1H, Py-H), 7.30 (d, 1H, pyrimidyl-H), 6.75 (d, 1H, Ar–H), 6.70 (d, 1H, Ar–H), 6.38 (dd, 1H, Ar–H), 2.08 (s, 3H, –CH_3_). IR (KBr, cm^−1^): 3076, 1655, 1521, 1476, 870. MS (ESI) *m/z*: 308.11.

#### General procedure for the synthesis of 6-methyl-*N*^1^-(4-(pyridin-3-yl)pyrimidin-2-yl)benzene-1,3-diamine (**6**)

To a solution of stannous chloride dihydrate in hydrochloric acid (30 mL) at 0 °C, *N*-(2-methyl-5-nitrophenyl)-4-pyridin-3-yl-pyrimidin-2-ylamine **5** was added in portions and stirred for 6 h. Progress of reaction was monitored by TLC. Upon completion, the mixture was poured into crushed ice, made alkaline with solid sodium hydroxide, and extracted with ethyl acetate. The combined organic layer was washed two to three times with water and dried over anhydrous sodium sulfate. The solvent was evaporated to get crude product, which was purified by recrystallization from methylene chloride to get the compound as a yellow solid.

#### Synthesis of 6-methyl-*N*^1^-(4-(pyridin-3-yl)pyrimidin-2-yl)benzene-1,3-diamine **(6)**

The product obtained was yellow solid (75%) from *N*-(2-methyl-5-nitrophenyl)-4-pyridin-3-yl-pyrimidin-2-ylamine **5** (10 g, 3.254 mmol), and stannous chloride dihydrate (29 g, 12.974 mmol) in 35 mL hydrochloric acid. MP: 142–144 °C. ^1^H-NMR δ: 8.98 (d, 1H, Py-H), 8.65 (dd, 1H, Py-H), 8.58 (s, 1H, –NH), 8.42 (d, 1H, pyrimidyl-H), 8.34 (d, 1H, Py-H), 7.48 (dd, 1H, Py-H), 7.30 (d, 1H, pyrimidyl-H), 6.82 (d, 1H, Ar–H), 6.75 (d, 1H, Ar–H), 6.30 (dd, 1H, Ar–H), 4.80 (br, 2H, –NH_2_), 2.05 (s, 3H, –CH_3_). MS (ESI) *m/z*: 278.13.

#### General procedure for the synthesis of *N*-(4-methyl-3-((4-(pyridin-3-yl)pyrimidin-2-yl)amino)phenyl)piperidine-4-carboxamide (**8**)

Piperidine-4-carboxylic acid **7** was taken in dry *N,N*-dimethyl formamide and cooled to 0–5 °C in ice bath. Then isobutyl chloroformate and *N*-methyl morpholine were added to the reaction mixture. The reaction mixture was allowed to stir for 10–15 min. After that 6-methyl-*N*1-(4-(pyridin-3-yl)pyrimidin-2-yl)benzene-1,3-diamine **6** was added, then reaction mixture was allowed to room temperature under stirring for 5–6 h. Progress of reaction was monitored by TLC. Upon completion, the solvent was removed under reduced pressure and residue was taken in water and extracted with ethyl acetate. The organic layer was dried with anhydrous sodium sulphate, the solvent was evaporated to get crude product which was purified by column chromatography over silica gel (60–120 mesh) using MDC and methanol (1:1) to get *N*-(4-methyl-3-((4-(pyridin-3-yl)pyrimidin-2-yl)amino)phenyl)piperidine-4-carboxamide (**8**).

#### Synthesis of *N*-(4-methyl-3-((4-(pyridin-3-yl)pyrimidin-2-yl)amino)phenyl)piperidine-4-carboxamide (**8**)

The product obtained was pale yellow color from piperidine-4-carboxylic acid **7** (0.046 g, 0.36 mmol), 6-methyl-*N*1-(4-(pyridin-3-yl)pyrimidin-2-yl)benzene-1,3-diamine **6** (0.1 g, 0.36 mmol), isobutyl chloroformate (0.078 g, 0.772 mmol) and *N*-methyl morpholine (0.078 g, 0.772 mmol). MP: 118–120 °C. ^1^H-NMR δ: 9.20 (s, 1H, –CO–NH), 8.95 (d, 1H, Py-H), 8.70 (dd, 1H, Py-H), 8.61 (s, 1H, –NH), 8.46 (d, 1H, pyrimidyl-H), 8.32 (d, 1H, Py-H), 7.40 (dd, 1H, Py-H), 7.33 (d, 1H, pyrimidyl-H), 6.76 (d, 1H, Ar–H), 6.69 (d, 1H, Ar–H), 6.32 (dd, 1H, Ar–H), 3.53 (t, 2H, –CH_2_), 3.28 (s, 1H, –NH), 3.20 (t, 2H, –CH_2_), 2.79-2.89 (bs, 1H, –CH), 2.35 (t, 2H, –CH_2_), 2.10 (t, 2H, –CH_2_), 2.01 (s, 3H, –CH_3_). MS (ESI) *m/z*: 389.2 (100.0%). Anal. calcd. for C_22_H_24_N_6_O (in %): C-68.02, H-6.23, N-21.63. Found: C-67.96, H-6.17, N-21.65.

#### General procedure for the synthesis of *N*-(4-methyl-3-((4-(pyridin-3-yl)pyrimidin-2-yl)amino)phenyl)piperidine-4-carboxamide derivatives **10**(**a**–**f**)

The *N*-(4-methyl-3-((4-(pyridin-3-yl)pyrimidin-2-yl)amino)phenyl)piperidine-4-carboxamide (**8**) was dissolved in dry dichloromethane. To this reaction mixture triethylamine was added and cooled to 0–5 °C in ice bath. Then different sulfonyl chlorides **9**(**a**–**f**) are added. The reaction mixture was monitored by TLC. Upon completion, the solvent was removed under reduced pressure and residue was taken in water and extracted with ethyl acetate. The organic layer was dried with anhydrous sodium sulphate and the solvent was evaporated to get crude product which was purified by column chromatography over silica gel (60–120 mesh) using dichloromethane and methanol (1:1).

#### Synthesis of *N*-(4-methyl-3-((4-(pyridin-3-yl)pyrimidin-2-yl)amino)phenyl)-1-((4-nitrophenyl) sulfonyl)piperidine-4-carboxamide (**10a**)

The product obtained was pale yellow color from *N*-(4-methyl-3-((4-(pyridin-3-yl)pyrimidin-2-yl)amino)phenyl)piperidine-4-carboxamide (**8**) (0.1 g, 0.257 mmol), 4-nitrobenzene sulfonyl chloride (**9a**) (0.055 g, 0.257 mmol) and triethylamine (0.078 g, 0.772 mmol). ^1^H-NMR δ: 9.23 (s, 1H, –CO–NH), 8.92 (d, 1H, Py-H), 8.75 (dd, 1H, Py-H), 8.60 (s, 1H, -NH), 8.47 (d, 1H, pyrimidyl-H), 8.40 (d, 2H, Ar–H), 8.30 (d, 1H, Py-H), 8.15 (d, 2H, Ar–H), 7.43 (dd, 1H, Py-H), 7.30 (d, 1H, pyrimidyl-H), 6.71 (d, 1H, Ar–H), 6.69 (d, 1H, Ar–H), 6.35 (dd, 1H, Ar–H), 3.50 (t, 2H, –CH_2_), 3.25 (t, 2H, –CH_2_), 2.80–2.88 (bs, 1H, –CH), 2.38 (t, 2H, –CH_2_), 2.12 (t, 2H, –CH_2_), 2.03 (s, 3H, –CH_3_). ^13^C NMR (100.6 MHz, DMSO-*d*_6_) δ: 17.5, 29.1, 38.1, 46.3, 103.3, 108.1, 111.7, 123.9, 124.2, 124.8, 128.3, 130.0, 133.1, 134.2, 136.3, 142.2, 145.8, 147.4, 148.0, 151.3, 154.5, 161.1, 168.7, 172.9. MS (ESI) *m/z*: 574.18 (100.0%). Anal. calcd. for C_28_H_27_N_7_O_5_S (in %): C-58.63, H-4.74, N-17.09. Found: C-58.66, H-4.71, N-17.05.

#### Synthesis of *N-*(4-methyl-3-((4-(pyridin-3-yl)pyrimidin-2-yl)amino)phenyl)-1-(o-tolylsulfonyl)piperidine-4-carboxamide (**10b**)

The product obtained was pale yellow color from *N*-(4-methyl-3-((4-(pyridin-3-yl)pyrimidin-2-yl)amino)phenyl)piperidine-4-carboxamide **(8)** (0.1 g, 0.257 mmol), 2-methylbenzene sulfonyl chloride **(9b)** (0.049 g, 0.257 mmol) and triethylamine(0.078 g, 0.772 mmol). ^1^H-NMR δ: 9.23 (s, 1H, –CO–NH), 8.91 (d, 1H, Py-H), 8.73 (dd, 1H, Py-H), 8.60 (s, 1H, -NH), 8.49 (d, 1H, pyrimidyl-H), 8.40 (d, 1H, Py-H), 7.78 (d, 1H, Ar–H), 7.53 (d, 1H, Ar–H), 7.45 (t, 2H, Ar–H), 7.37 (dd, 1H, Py-H), 7.28 (d, 1H, pyrimidyl-H), 6.73 (d, 1H, Ar–H), 6.60 (d, 1H, Ar–H), 6.35 (dd, 1H, Ar–H), 3.50 (t, 2H, –CH_2_), 3.25 (t, 2H, –CH_2_), 2.78-2.85 (bs, 1H, –CH), 2.70 (s, 3H, –CH_3_), 2.32 (t, 2H, –CH_2_), 2.15 (t, 2H, –CH_2_), 2.05 (s, 3H, –CH_3_). MS (ESI) *m/z*: 543.21 (100.0%). Anal. calcd. for C_29_H_30_N_6_O_3_S (in %): C-64.19, H-5.57, N-15.49. Found: C-64.16, H-5.51, N-15.45.

#### Synthesis of 1-((4-methoxyphenyl)sulfonyl)-*N-*(4-methyl-3-((4-(pyridin-3-yl)pyrimidin-2-yl)amino)phenyl)piperidine-4-carboxamide (**10c**)

The product obtained was pale yellow color from *N*-(4-methyl-3-((4-(pyridin-3-yl)pyrimidin-2-yl)amino)phenyl)piperidine-4-carboxamide (**8**) (0.1 g, 0.257 mmol), 4-methoxybenzene sulfonyl chloride (**9c**) (0.053 g, 0.257 mmol) and triethylamine(0.078 g, 0.772 mmol). ^1^H-NMR δ: 9.18 (s, 1H, –CO–NH), 8.90 (d, 1H, Py-H), 8.74 (dd, 1H, Py-H), 8.65 (s, 1H, –NH), 8.40 (d, 1H, pyrimidyl-H), 8.35 (d, 1H, Py-H), 7.63 (dd, 2H, Ar–H), 7.35 (dd, 1H, Py-H), 7.30 (d, 1H, pyrimidyl-H), 7.10 (d, 2H, Ar–H), 6.70 (d, 1H, Ar–H), 6.63 (d, 1H, Ar–H), 6.30 (dd, 1H, Ar–H), 3.84 (s, 3H, –OCH_3_), 3.50 (t, 2H, –CH_2_), 3.24 (t, 2H, –CH_2_), 2.80–2.88 (bs, 1H, –CH), 2.34 (t, 2H, –CH_2_), 2.11 (t, 2H, –CH_2_), 2.03 (s, 3H, –CH_3_). MS (ESI) *m/z*: 559.20 (100.0%), Anal. calcd. for C_29_H_30_N_6_O_4_S (in %): C-62.35, H-5.41, N-15.04. Found: C-62.29, H-5.37, N-15.05.

#### Synthesis of 1-((3-chlorophenyl)sulfonyl)-*N-*(4-methyl-3-((4-(pyridin-3-yl)pyrimidin-2-yl)amino)phenyl)piperidine-4-carboxamide (**10d**)

The product obtained was pale yellow color from *N*-(4-methyl-3-((4-(pyridin-3-yl)pyrimidin-2-yl)amino)phenyl)piperidine-4-carboxamide (**8**) (0.1 g, 0.257 mmol) and 3-chlorobenzene sulfonyl chloride (**9d**) (0.054 g, 0.257 mmol) and triethylamine(0.078 g, 0.772 mmol). ^1^H-NMR δ: 9.21 (s, 1H, –CO–NH), 8.95 (d, 1H, Py-H), 8.74 (dd, 1H, Py-H), 8.60 (s, 1H, –NH), 8.51 (d, 1H, pyrimidyl-H), 8.36 (d, 1H, Py-H), 8.21 (s, 1H, Ar–H), 7.75 (d, 1H, Ar–H), 7.66 (t, 1H, Ar–H), 7.51 (d, 1H, Ar–H), 7.42 (dd, 1H, Py-H), 7.31 (d, 1H, pyrimidyl-H), 6.74 (d, 1H, Ar–H), 6.67 (d, 1H, Ar–H), 6.30 (dd, 1H, Ar–H), 3.55 (t, 2H, –CH_2_), 3.22 (t, 2H, –CH_2_), 2.79–2.89 (bs, 1H, –CH), 2.36 (t, 2H, –CH_2_), 2.12 (t, 2H, –CH_2_), 2.05 (s, 3H, –CH_3_). MS (ESI) *m/z*: 563.29 (100.0%). Anal. calcd. for C_28_H_27_ClN_6_O_3_S (in %): C-59.73, H-4.83, N-14.93. Found: C- C-59.70, H-4.81, N-14.90.

#### Synthesis of 1-((3,4-difluorophenyl)sulfonyl)-*N-*(4-methyl-3-((4-(pyridin-3-yl)pyrimidin-2-yl)amino)phenyl)piperidine-4-carboxamide (**10e**)

The product obtained was dark brown color from *N*-(4-methyl-3-((4-(pyridin-3-yl)pyrimidin-2-yl)amino)phenyl)piperidine-4-carboxamide (**8**) (0.1 g, 0.257 mmol) and 3,4-difluorobenzene sulfonyl chloride (**9e**) (0.054 g, 0.257 mmol) and triethylamine(0.078 g, 0.772 mmol). ^1^H-NMR δ: 9.19 (s, 1H, –CO–NH), 8.92 (d, 1H, Py-H), 8.75 (dd, 1H, Py-H), 8.66 (s, 1H, –NH), 8.42 (d, 1H, pyrimidyl-H), 8.30 (d, 1H, Py-H), 7.89 (s, 1H, Ar–H), 7.73 (dd, 1H, Ar–H), 7.49 (dd, 1H, Ar–H), 7.41 (dd, 1H, Py-H), 7.30 (d, 1H, pyrimidyl-H), 6.78 (d, 1H, Ar–H), 6.62 (d, 1H, Ar–H), 6.36 (dd, 1H, Ar–H), 3.50 (t, 2H, –CH_2_), 3.20 (t, 2H, –CH_2_), 2.75-2.83 (bs, 1H, –CH), 2.30 (t, 2H, –CH_2_), 2.15 (t, 2H, –CH_2_), 2.00 (s, 3H, –CH_3_). MS (ESI) *m/z*: 565.17 (100.0%), Anal. calcd. for C_28_H_26_F_2_N_6_O_3_S (in %): C-59.56, H-4.64, N-14.88. Found: C-59.50, H-4.61, N-14.92.

#### Synthesis of 1-((2,6-difluorophenyl)sulfonyl)-*N*-(4-methyl-3-((4-(pyridin-3-yl)pyrimidin-2-yl)amino)phenyl)piperidine-4-carboxamide (**10f**)

The product obtained was dark brown color from *N*-(4-methyl-3-((4-(pyridin-3-yl)pyrimidin-2-yl)amino)phenyl)piperidine-4-carboxamide (**8**) (0.1 g, 0.257 mmol) and 2,6-difluorobenzene sulfonyl chloride (**9f**) (0.054 g, 0.257 mmol) and triethylamine (0.078 g, 0.772 mmol). ^1^H-NMR δ: 9.22 (s, 1H, –CO–NH), 8.98 (d, 1H, Py-H), 8.68 (dd, 1H, Py-H), 8.59 (s, 1H, –NH), 8.47 (d, 1H, pyrimidyl-H), 8.35 (d, 1H, Py-H), 7.45 (dd, 1H, Py-H), 7.36 (d, 1H, pyrimidyl-H), 7.25 (dd, 2H, Ar–H), 7.19 (t, 1H, Ar–H), 6.80 (d, 1H, Ar–H), 6.71 (d, 1H, Ar–H), 6.36 (dd, 1H, Ar–H), 3.57 (t, 2H, –CH_2_), 3.22 (t, 2H, –CH_2_), 2.78–2.88 (bs, 1H, –CH), 2.32 (t, 2H, –CH_2_), 2.15 (t, 2H, –CH_2_), 2.04 (s, 3H, –CH_3_). MS (ESI) *m/z*: 565.17 (100.0%). Anal. calcd. for C_28_H_26_F_2_N_6_O_3_S (in %): C-59.56, H-4.64, N-14.88. Found: C-59.52, H-4.60, N-14.90.

#### Synthesis of 1-(4-chlorobenzoyl)-*N-*(4-methyl-3-((4-(pyridin-3-yl)pyrimidin-2-yl)amino)phenyl)piperidine-4-carboxamide (**12a**)

The product obtained was dark brown color from *N*-(4-methyl-3-((4-(pyridin-3-yl)pyrimidin-2-yl)amino)phenyl)piperidine-4-carboxamide (**8**) (0.1 g, 0.257 mmol) and 4-chlorobenzoyl chloride (**11a**) (0.045 g, 0.257 mmol) and triethylamine(0.078 g, 0.772 mmol). ^1^H-NMR δ: 9.18 (s, 1H, –CO–NH), 8.90 (d, 1H, Py-H), 8.75 (dd, 1H, Py-H), 8.66 (s, 1H, –NH), 8.50 (d, 1H, pyrimidyl-H), 8.34 (d, 1H, Py-H), 7.82 (dd, 2H, Ar–H), 7.65 (dd, 2H, Ar–H), 7.40 (dd, 1H, Py-H), 7.33 (d, 1H, pyrimidyl-H), 6.72 (d, 1H, Ar–H), 6.65 (d, 1H, Ar–H), 6.38 (dd, 1H, Ar–H), 3.50 (t, 2H, –CH_2_), 3.23 (t, 2H, –CH_2_), 2.78–2.87 (bs, 1H, –CH), 2.37 (t, 2H, –CH_2_), 2.11 (t, 2H, –CH_2_), 2.02 (s, 3H, –CH_3_). ^13^C NMR (100.6 MHz, DMSO-*d*_6_) δ: 17.6, 29.7, 38.3, 44.7, 103.5, 108.1, 111.5, 123.8, 124.7, 128.7, 129.6, 133.0, 134.1, 135.3, 136.3, 142.2, 147.5, 148.0, 154.5, 161.1, 168.7, 170.0, 172.9. MS (ESI) *m/z*: 527.018 (100.0%), Anal. calcd. for C_29_H_27_ClN_6_O_2_ (in %): C-66.09, H-5.16, N-15.95. Found: C-66.05, H-5.13, N-15.92.

#### Synthesis of 1-(4-fluorobenzoyl)-*N*-(4-methyl-3-((4-(pyridin-3-yl)pyrimidin-2-yl)amino)phenyl)piperidine-4-carboxamide (**12b**)

The product obtained was dark brown color from *N*-(4-methyl-3-((4-(pyridin-3-yl)pyrimidin-2-yl)amino)phenyl)piperidine-4-carboxamide (**8**) (0.1 g, 0.257 mmol) and 4-fluorobenzoyl chloride (**11b**) (0.040 g, 0.257 mmol) and triethylamine(0.078 g, 0.772 mmol). ^1^H-NMR δ: 9.20 (s, 1H, –CO–NH), 8.93 (d, 1H, Py-H), 8.79 (dd, 1H, Py-H), 8.61 (s, 1H, -NH), 8.55 (d, 1H, pyrimidyl-H), 8.30 (d, 1H, Py-H), 7.80 (dd, 2H, Ar–H), 7.65 (dd, 2H, Ar–H), 7.44 (dd, 1H, Py-H), 7.30 (d, 1H, pyrimidyl-H), 6.74 (d, 1H, Ar–H), 6.60 (d, 1H, Ar–H), 6.42 (dd, 1H, Ar–H), 3.55 (t, 2H, –CH_2_), 3.28 (t, 2H, –CH_2_), 2.76–2.87 (bs, 1H, –CH), 2.35 (t, 2H, –CH_2_), 2.14 (t, 2H, –CH_2_), 2.01 (s, 3H, –CH_3_). MS (ESI) *m/z*: 511.21 (100.0%), Anal. calcd. for C_29_H_27_FN_6_O_2_ (in %): C-68.22, H-5.33, N-16.46. Found: C-68.20, H-5.29, N-16.41.

#### Synthesis of *N*-(4-methyl-3-((4-(pyridin-3-yl)pyrimidin-2-yl)amino)phenyl)-1-(4-(trifluoromethyl)benzoyl)piperidine-4-carboxamide (**12c**)

The product obtained was dark brown color from *N*-(4-methyl-3-((4-(pyridin-3-yl)pyrimidin-2-yl)amino)phenyl)piperidine-4-carboxamide (**8**) (0.1 g, 0.257 mmol) and 4-(trifluoromethyl)benzoyl chloride (**11c**) (0.053 g, 0.257 mmol) and triethylamine(0.078 g, 0.772 mmol). ^1^H-NMR δ: 9.20 (s, 1H, –CO–NH), 8.93 (d, 1H, Py-H), 8.81 (dd, 1H, Py-H), 8.65 (s, 1H, –NH), 8.52 (d, 1H, pyrimidyl-H), 8.34 (d, 1H, Py-H), 7.98 (dd, 2H, Ar–H), 7.86 (dd, 2H, Ar–H), 7.50 (dd, 1H, Py-H), 7.36 (d, 1H, pyrimidyl-H), 6.74 (d, 1H, Ar–H), 6.63 (d, 1H, Ar–H), 6.40 (dd, 1H, Ar–H), 3.53 (t, 2H, –CH_2_), 3.25 (t, 2H, –CH_2_), 2.74-2.85 (bs, 1H, –CH), 2.32 (t, 2H, –CH_2_), 2.14 (t, 2H, –CH_2_), 2.03 (s, 3H, –CH_3_). MS (ESI) *m/z*: 561.21 (100.0%), Anal. calcd. for C_30_H_27_F_3_N_6_O_2_ (in %): C-64.28, H-4.85, N-14.99. Found: C-64.22, H-4.80, N-14.93.

#### Synthesis of 1-((4-chlorophenyl)carbamothioyl)-*N*-(4-methyl-3-((4-(pyridin-3-yl)pyrimidin-2-yl)amino)phenyl)piperidine-4-carboxamide (**14a**)

The product obtained was dark brown color from *N*-(4-methyl-3-((4-(pyridin-3-yl)pyrimidin-2-yl)amino)phenyl)piperidine-4-carboxamide (**8**) (0.1 g, 0.257 mmol) and 4-chlorophenyl isothiocyanate (**13a**) (0.043 g, 0.257 mmol) and triethylamine(0.078 g, 0.772 mmol). ^1^H-NMR δ: 9.22 (s, 1H, –CO–NH), 9.16 (s, 1H, –CS–NH), 8.93 (d, 1H, Py-H), 8.74 (dd, 1H, Py-H), 8.59 (s, 1H, –NH), 8.42 (d, 1H, pyrimidyl-H), 8.30 (d, 1H, Py-H), 7.46 (dd, 1H, Py-H), 7.35 (d, 1H, pyrimidyl-H), 7.29 (dd, 2H, Ar–H), 6.60 (dd, 2H, Ar–H), 6.71 (d, 1H, Ar–H), 6.62 (d, 1H, Ar–H), 6.35 (dd, 1H, Ar–H), 3.50 (t, 2H, –CH_2_), 3.23 (t, 2H, –CH_2_), 2.79–2.89 (bs, 1H, –CH), 2.33 (t, 2H, –CH_2_), 2.11 (t, 2H, –CH_2_), 2.03 (s, 3H, –CH_3_). ^13^C NMR (100.6 MHz, DMSO-*d*_6_) δ: 17.6, 29.7, 38.3, 51.0, 103.5, 108.1, 111.5, 123.8, 124.7, 128.7, 129.6, 131.7, 133.0, 133.7, 134.1, 136.3, 142.2, 147.7, 148.0, 154.5, 161.1, 168.5, 172.9, 186.7. MS (ESI) *m/z*: 557.17 (100.0%), Anal. calcd. for C_29_H_28_ClN_7_OS (in %): C-62.41, H-5.06, N-17.57. Found: C-62.37, H-5.01, N-17.53.

#### Synthesis of 1-((2-methoxyphenyl)carbamothioyl)-*N*-(4-methyl-3-((4-(pyridin-3-yl)pyrimidin-2-yl)amino)phenyl)piperidine-4-carboxamide (**14b**)

The product obtained was dark brown color color from *N*-(4-methyl-3-((4-(pyridin-3-yl)pyrimidin-2-yl)amino)phenyl)piperidine-4-carboxamide (**8**) (0.1 g, 0.257 mmol) and 2-methoxyphenyl isothiocyanate (**13b**) (0.042 g, 0.257 mmol) and triethylamine(0.078 g, 0.772 mmol). ^1^H-NMR δ: 9.20 (s, 1H, –CO–NH), 9.14 (s, 1H, –CS–NH), 8.92 (d, 1H, Py-H), 8.75 (dd, 1H, Py-H), 8.63 (s, 1H, –NH), 8.40 (d, 1H, pyrimidyl-H), 8.35 (d, 1H, Py-H), 7.42 (dd, 1H, Py-H), 7.31 (d, 1H, pyrimidyl-H), 6.86 (d, 1H, Ar–H), 6.79 (d, 1H, Ar–H), 6.70 (dd, 2H, Ar–H), 6.68 (d, 1H, Ar–H), 6.59 (d, 1H, Ar–H), 6.34 (dd, 1H, Ar–H), 3.85 (s, 3H, –OCH_3_), 3.56 (t, 2H, –CH_2_), 3.24 (t, 2H, –CH_2_), 2.75–2.86 (bs, 1H, –CH), 2.37 (t, 2H, –CH_2_), 2.14 (t, 2H, –CH_2_), 2.05 (s, 3H, –CH_3_). MS (ESI) *m/z*: 554.22 (100.0%), Anal. calcd. for C_30_H_31_N_7_O_2_S (in %): C-65.08, H-5.64, N-17.71. Found: C-65.02, H-5.60, N-17.66.

#### Synthesis of 1-((3-methoxyphenyl)carbamothioyl)-*N*-(4-methyl-3-((4-(pyridin-3-yl)pyrimidin-2-yl)amino)phenyl)piperidine-4-carboxamide (**14c**)

The product obtained was dark brown color from *N*-(4-methyl-3-((4-(pyridin-3-yl)pyrimidin-2-yl)amino)phenyl)piperidine-4-carboxamide (**8**) (0.1 g, 0.257 mmol) and 3-methoxyphenyl isothiocyanate (**13c**) (0.042 g, 0.257 mmol) and triethylamine(0.078 g, 0.772 mmol). ^1^H-NMR δ: 9.21 (s, 1H, –CO–NH), 9.15 (s, 1H, –CS–NH), 8.90 (d, 1H, Py-H), 8.75 (dd, 1H, Py-H), 8.64 (s, 1H, –NH), 8.42 (d, 1H, pyrimidyl-H), 8.36 (d, 1H, Py-H), 7.48 (dd, 1H, Py-H), 7.34 (d, 1H, pyrimidyl-H), 7.10 (t, 1H, Ar–H), 6.78 (d, 1H, Ar–H), 6.62 (d, 1H, Ar–H), 6.38 (d, 1H, Ar–H), 6.30 (dd, 1H, Ar–H), 6.25 (bs, 1H, Ar–H), 6.08 (d, 1H, Ar–H), 3.83 (s, 3H, –OCH_3_), 3.54 (t, 2H, –CH_2_), 3.25 (t, 2H, –CH_2_), 2.79–2.89 (bs, 1H, –CH), 2.38 (t, 2H, –CH_2_), 2.15 (t, 2H, –CH_2_), 2.01 (s, 3H, –CH_3_). MS (ESI) *m/z*: 554.226 (100.0%), Anal. calcd. for C_30_H_31_N_7_O_2_S (in %): C-65.08, H-5.64, N-17.71. Found: C-65.03, H-5.60, N-17.73.

### Biology

Fertilized eggs were obtained from IVRI, Bangalore, India. CT DNA was purchased from Sigma. All chemicals and solvents were reagent grade purchased from Merck. DNA stock solution was prepared by dilution of CT DNA to buffer (containing 150 mM NaCl and 15 mM trisodium citrate at pH 7.0) followed by exhaustive stirring at 4 °C for 3 days, and kept at 4 °C for no longer than a week. The stock solution of CT DNA gave a ratio of UV absorbance at 260 and 280 nm (A260/A280) of 1.89, indicating that the DNA was sufficiently free of protein contamination. The DNA concentration was determined by the UV absorbance at 260 nm after 1:20 dilution using ε = 6600 M^−1^ cm^−1^.

#### Shell less chorioallantoic membrane (CAM) assay

Antiangiogenic effect of the novel *N*-(4-methyl-3-((4-(pyridin-3-yl)pyrimidin-2-yl)amino)phenyl)piperidine-4-carboxamide derivatives **10**(**a**–**f**), **12**(**a**–**c**) and **14**(**a**–**c**) was evaluated according to the method of Auerbach et al. [38]. Fertilized hens eggs were surface sterilized using 70% alcohol. The eggs were incubated in fan assisted humidified incubator at 37 °C. On the 4th day, the eggs were cracked out into thin films of the hammock within a laminar flow cabinet and were further incubated. On the day 5th when blood vessels were seen proliferating from the center of the eggs within the hammock, filter paper discs loaded with 100 µg of **10**(**a**–**f**), **12**(**a**–**c**) and **14**(**a**–**c**) were placed over the proliferating blood vessels and the eggs were returned to the incubator. Results for antiangiogenic effect of the each compound were observed after 24 h comparing to untreated controls (paper discs with solvent only).

#### DNA cleavage experiments

DNA cleavage experiments were carried out according to the previously described procedure [39]. Briefly, the solution of compounds in DMF (1 mg/mL) was prepared and these test samples (1 µg) were added to the 500 ng of Calf thymus-DNA (CT-DNA) in TE buffer and incubated for 2 h at 37 °C. Agarose gel electrophoresis was performed after loading the samples on to the gel in TAE buffer system at 50 V for 2 h. At the end of electrophoresis, the gel was carefully stained with EtBr (Ethedium bromide) solution (10 µg/mL) for 10–15 min and visualized under UV light using a Bio-Rad Trans illuminator and the images were captured.

## Results and discussions

### Chemistry

**Scheme 1:**
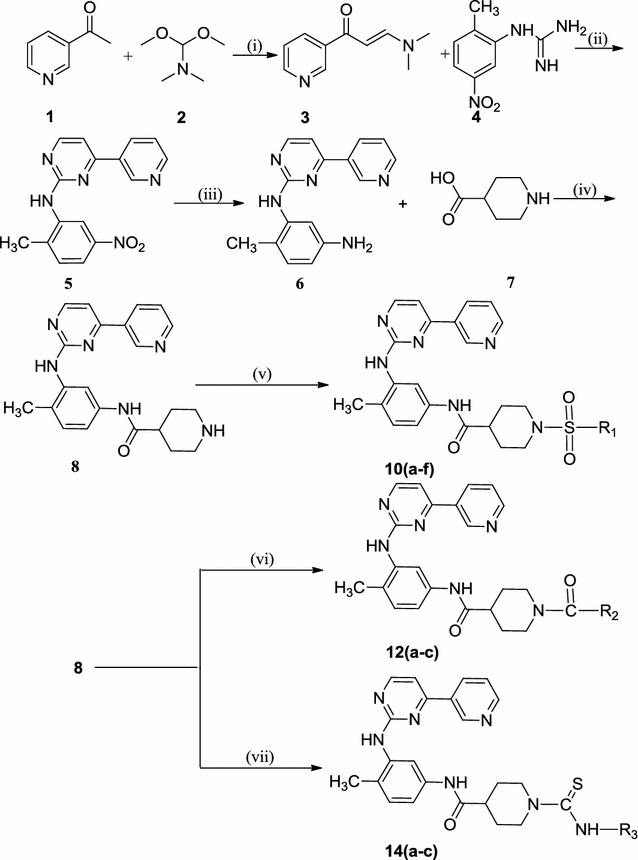
Synthesis of *N*-(4-methyl-3-((4-(pyridin-3-yl)pyrimidin-2-yl)amino)phenyl)piperidine-4-carboxamide and its analogs. Reagents and conditions: (i) 100 °C; (ii) NaOH, *n*-butanol, 110 °C; (iii) SnCl_2_·2H_2_O, hydrochloric acid, 0 °C-r.t.; (iv) IBCF, NMP, DMF; (v) MDC, TEA, 0 °C-r.t. **9a** = 4-nitrobenzene sulfonyl chloride; **9b** = 2-methylbenzene sulfonyl chloride; **9c** = 4-methoxybenzene sulfonyl chloride; **9d** = 3-chlorobenzene sulfonyl chloride; **9e** = 3,4-difluorobenzene sulfonyl chloride; **9f** = 2,6-difluorobenzene sulfonyl chloride; (vi) MDC, TEA, 0 0C-r.t. 11a = 4-chlorobenzoyl chloride; **11b** = 4-fluorobenzoyl chloride; **11c** = 4-(trifluoromethyl)benzoyl chloride; (vii) MDC, TEA, 0 0C-r.t; **13a** = 4-chlorophenyl isothiocyanate; **13b** = 2-methoxyphenyl isothiocyanate; **13c** = 3-methoxyphenyl isothiocyanate

Synthesis of the key intermediate *N*-(4-methyl-3-((4-(pyridin-3-yl)pyrimidin-2-yl)amino)phenyl)piperidine-4-carboxamide (**8**) is outlined in Scheme 1. To prepare the pyrimidine ring system, the general method was used [40], which involved reacting 3-acetylpyridine (**1**) with *N*,*N*-dimethylformamide dimethyl acetyl (**2**) to give the 3-dimethylamino-1-(pyridin-3-yl)prop-2-en-1-one (**3**) in 90% yield. The enaminone (**3**) reacts with 1-(2-methyl-5-nitrophenyl)guanidine (**4**) in presence of base to give *N*-(2-methyl-5-nitrophenyl)-4-(pyridin-3-yl)pyrimidin-2-amine (**5**). Reduction of compound (**5**) with SnCl_2_·2H_2_O afforded 6-methyl-N1-(4-pyridin-3-yl-pyrimidin-2-yl)benzene-1,3-diamine (**6**) in 75% yield. 6-Methyl-N1-(4-(pyridin-3-yl)pyrimidin-2-yl)benzene-1,3-diamine (**6**) (1.0 eq) and piperidine-4-carboxylic acid (**7**) (1.0 eq) in *N*,*N*-dimethyl formamide in the presence of base *N*-methyl morpholine, isobutyl chloroformate, and reaction mixture was stirred for 5–6 h at room temperature, which gave target key intermediate (**8**). The absence of –COOH proton peak and presence of –NH proton peak confirmed the formation of compound (**8**) with a good yield of 88%. The nucleophilic substitution reaction of *N*-(4-methyl-3-((4-(pyridin-3-yl)pyrimidin-2-yl)amino)phenyl)piperidine-4-carboxamide (**8**) with different substituted aromatic sulfonyl chlorides** 9**(**a**–**f**) (R–SO_2_Cl)/aromatic acid chlorides** 11**(**a**–**c**) (R–CO–Cl)/aromatic isothiocyanates** 13**(**a**–**c**) (R–N=C=S) was carried out in the presence of triethylamine and dichloromethane as solvent with a good yield of 81–88%. The absence of –NH and presence of –CS–NH proton peak in synthesized derivatives** 10**(**a**–**f**),** 12**(**a**–**c**) and** 14**(**a**–**c**) in 1H NMR spectra confirmed the identity of the products. It is also confirmed by IR data, for sulfonamide series 10(a–f) which showed asymmetric stretching frequency of O=S=O in the range 1350–1370 cm^−1^ and symmetric stretching frequency at 1270–1290 cm^−1^. For carboxamide series** 12**(**a**–**c**), IR data showed stretching frequency of –C=O at 1630–1670 cm^−1^ and similarly for** 14**(**a**–**c**), stretching frequency at 3350–3360 cm^−1^ for –NH and 1640–1660 cm^−1^ for –C=O group. The chemical structures of all the synthesized compounds are given in Table 1.

**Table 1:** Chemical structure, yield and melting point of the synthesized compounds

Compound	R_1_/R_2_/R_3_	Yield (%)	MP (°C)
**10a**		84	124
**10b**		81	135
**10c**		83	134
**10d**		85	145
**10e**		88	154
**10f**		86	153
**12a**		86	100
**12b**		85	88
**12c**		82	158
**14a**		86	143
**14b**		81	135
**14c**		82	136

### Biology

#### Choriallanotoic membrane (CAM) assay

The CAM assay is a simple, reliable, and inexpensive method of studying angiogenesis. In the present investigation anti-angiogenic activity of *N*-(4-methyl-3-((4-(pyridin-3-yl)pyrimidin-2-yl)amino)phenyl)piperidine-4-carboxamide derivatives showed reduced proliferation of blood vessels in the shell less CAM assay model of developing embryos.

Pyrimidine is an important scaffold known to be associated with several biological activities. Some of the derivatives of pyrimidines potently inhibit angiogenesis [41, 42]. Some representatives of pyrimidine have been investigated as non-ATP competitive KDR inhibitors (type II) [43]. Donnini et al. demonstrated that inhibition of pyrazolo-pyrimidine-derived c-Src kinase activity reduces VEGF induced-angiogenesis both in tumor and endothelial cells [44].

**Fig. 1:**
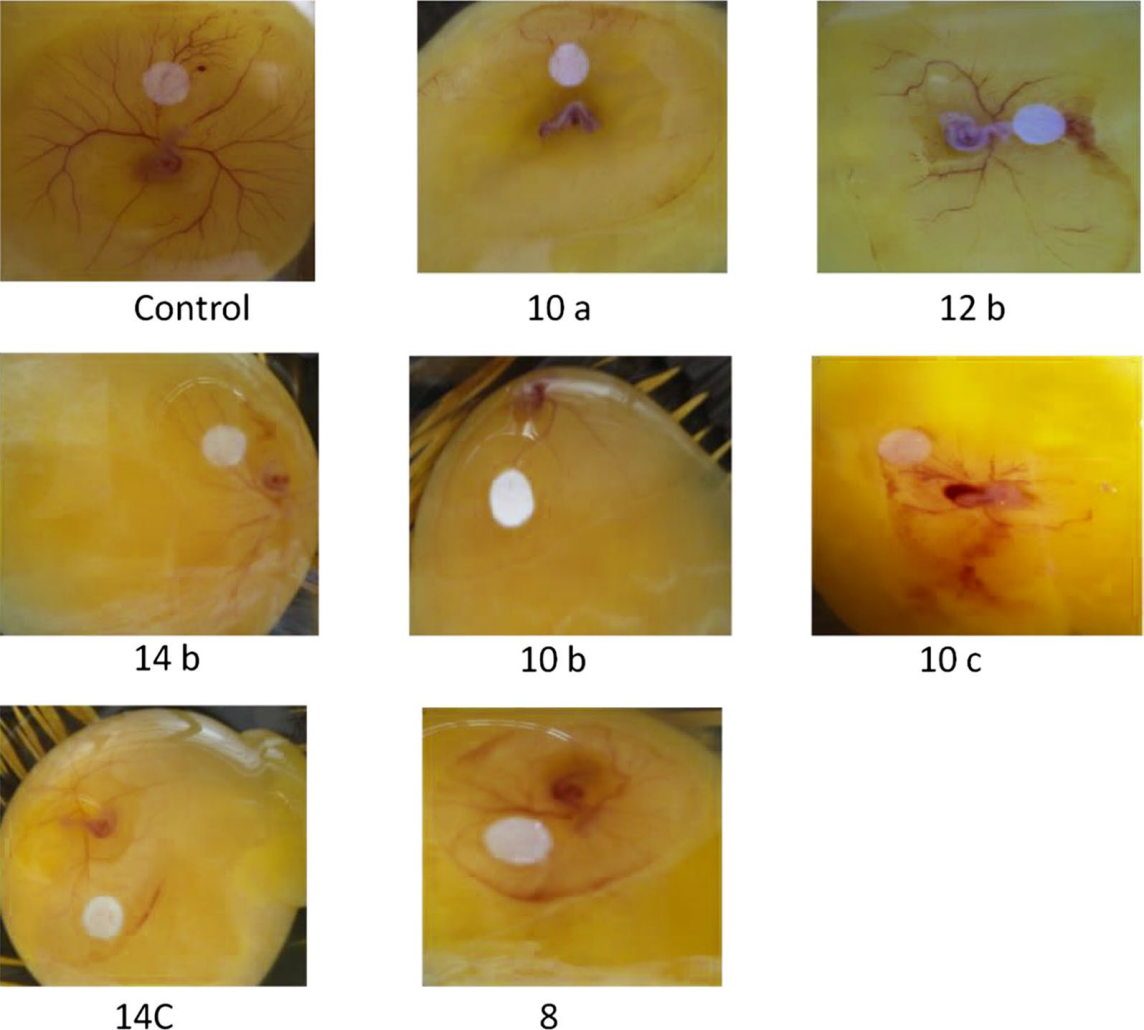
Suppression of angiogenesis in vivo by novel compounds **10a**,** 12b**, **14b**, **10b**, **10c** and **14c** in shell less CAM assay. Decreased vasculature was observed in treated groups compared to control

In view of the above findings, the anti-angiogenic activity was assessed by carrying out the reactions of *N*-(4-methyl-3-((4-(pyridin-3-yl)pyrimidin-2-yl)amino)phenyl)piperidine-4-carboxamide with different sulfonyl chlorides containing substituted aromatic rings. The proliferation of micro vessels were regressed around the zone of compounds treated (Fig. 1). Our data demonstrates that compounds **10a**, **10b**, **10c**, **12b**, **14b** and **14c** possess potential antiangiogenic activity.

#### DNA cleavage studies by gel electrophoresis

**Fig. 2:**
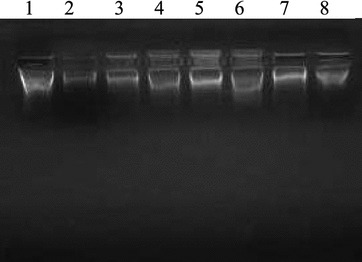
Photograph showing the effects of synthetic molecules on calf thymus DNA. Lane 1: Untreated DNA, lane 2: **10a**, lane 3: **10b**, lane 4: **10c**, lane 5: **10d**, lane 6: **14a**, lane7: **14b** lane 8: **14c**

**Fig. 3:**
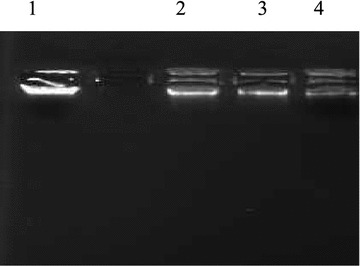
Photograph showing the effects of synthetic molecules on calf thymus DNA. Lane 1: Untreated DNA, lane 2:** 12a**, lane 3:** 12b**, lane 4: 8

**Fig. 4:**
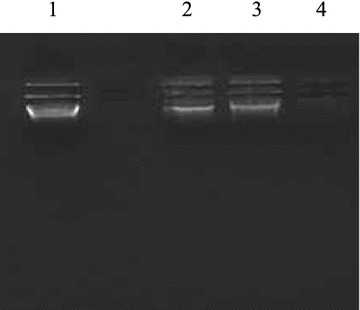
Photograph showing the effects of synthetic molecules on calf thymus DNA. Lane 1: Untreated DNA, lane 2: **12c**, lane 3: **10e**, lane 4: **10f**

The pyrimidine entity is one of the most prominent structures found in nucleic acid chemistry. Some of the derivatives of 4-(4-(6-phenyl-pyrimidin-4-yl)-phenoxymethyl]-chromen-2-ones were tested for DNA cleavage activity by agarose gel electrophoresis method [45]. Shamsuzzaman et al. synthesized some steroidal pyrimidines for interaction with DNA and indicated higher binding affinity of compounds towards DNA [46]. In view of the above findings, the compounds synthesized in this study were evaluated for their DNA cleavage activity. After binding to DNA, synthetic molecule can induce several changes in DNA conformation and deformations, such as bending, local denaturation, (over winding and under winding), intercalation, micro loop formation and subsequent DNA shortening lead to alteration in molecular weight of DNA. Gel electrophoresis is an extensively used technique for the study of binding of compounds with nucleic acids: in this method segregation of the molecules will be on the basis of their relative rate of movement through a gel under the influence of an electric field. Gel electrophoresis images shown in Figs. 2, 3 and 4 shows differences in band width and ethidium-bromide staining intensities compared to the control. The difference observed in the band width and intensity is the criterion for the evaluation of binding/cleavage ability of synthetic molecule with calf thymus DNA. Figure 2 shows the bands with different band width and brightness compared to control. There is significant binding/cleavage of DNA in the lane 2, 3, 4 and 6 when compared to the control, where the intensity of the DNA is more. Figure 3 shows lane 2, 3 and 4 (treated with synthetic molecule: **12a**, **12b**, and **8**) showed less intense DNA indicating degradation when compared with control. In the Fig. 4 lane 2, 3, 4 revealing less intense DNA compared to the control. The molecule 10f has completely degraded the DNA indicating better cleavage activity.

From the obtained results, it indicates that the substitution at N-terminal of the piperidine ring play a key role in its DNA binding activity. Thus,** 10b**,** 10c**,** 14b** and** 14c** having electron donating groups enhances their DNA binding/cleavage activity. Interestingly, compounds **10a** and **12b** having electron withdrawing nitro (para) and fluoro (ortho) groups, respectively also showed good activity. This could be attributed to the increased electron withdrawing effect of nitro and fluoro groups when compared to chloro group present in** 10**(**d**–**f**),** 12a**,** 12c** and** 14a**. On the other hand, as the electron donating efficiency increases, the activity also increases. We believe that introducing electron donating methoxy and methyl groups (**5e**,** 5f**) on the N-terminal of the piperidine ring at 2nd 3rd and 4th position resulted in increase in the activity. However, further studies are required to understand the exact mechanism of its action.

## Conclusion

Among the tested compounds, compounds **10a**, **10b**, **10c**, **12b**, **14b** and **14c** showed a significant antiangiogenic and DNA cleavage activity. *N*-(4-methyl-3-((4-(pyridin-3-yl)pyrimidin-2-yl)amino)phenyl)piperidine-4-carboxamide and its derivatives which showed combined antiangiogenic and DNA cleavage activities may be used for the design of more potent anticancer drugs. In conclusion, the antitumor activity of *N*-(4-methyl-3-((4-(pyridin-3-yl)pyrimidin-2-yl)amino)phenyl)piperidine-4-carboxamide and its analogs still has to be established, and detailed studies are needed to investigate whether these compounds are able to induce apoptosis in activated endothelial cells and in tumor vasculature.
